# Musculoskeletal injury epidemiology in law enforcement and firefighter recruits during physical training: a systematic review

**DOI:** 10.1136/bmjsem-2021-001289

**Published:** 2022-03-01

**Authors:** Myles Calder Murphy, Holly-Anne George, Muhammad Naqi, Patrick J Owen, Paola Chivers, Nicolas H Hart

**Affiliations:** 1 Institute for Nutrition Research, School of Medical and Health Sciences, Edith Cowan University, Joondalup, Western Australia, Australia; 2 School of Medical and Health Sciences, Edith Cowan University, Joondalup, Western Australia, Australia; 3 Institute for Physical Activity and Nutrition, Deakin University, Burwood, Victoria, Australia; 4 Institute of Health Research, The University of Notre Dame Australia, Fremantle, Western Australia, Australia; 5 College of Nursing and Health Sciences, Flinders University, Adelaide, South Australia, Australia

**Keywords:** epidemiology, injuries, public health

## Abstract

**Objectives:**

Report the injury epidemiology of law enforcement and firefighter recruits.

**Design:**

A systematic epidemiological review following the Preferred Reporting Items for Systematic Reviews and Meta-Analyses 2020 guidelines was completed.

**Data sources:**

Five online databases were searched from database inception to 5 May 2021.

**Eligibility criteria for selecting studies:**

Prospective and retrospective studies that reported data on musculoskeletal injuries sustained by law enforcement or firefighter recruits were included. We reported on all components of injury where data were available. All injury incidence rates were calculated as per 1000 training days (Poisson 95% CI) to allow comparisons between studies. Study quality was assessed using the Joanna Briggs Institute Quality Assessment Checklist for Prevalence Studies.

**Results:**

No studies reporting firefighter recruits were identified. Eight published studies that reported on injuries to law enforcement recruits were identified. The studies were all low quality, and the credibility of the evidence was assessed as very low. Seven studies reported medical attention injuries, and one study reported the number of medical withdrawals from a recruit training programme. The prevalence of law enforcement recruits with medical attention injuries ranged from 13.7% to 24.5%. The overall medical attention injury incidence rate for law enforcement recruits ranged from 1.67 injuries per 1000 training days (Poisson 95% CI 1.00 to 2.34 injuries per 1000 training days) to 4.24 injuries per 1000 training days (Poisson 95% CI 2.97 to 5.51 injuries per 1000 training days).

**Conclusion:**

This review reported the prevalence and incidence rates for musculoskeletal injuries in law enforcement officers. However, the credibility of the evidence is very low.

**PROSPERO registration number:**

CRD42021251084.

**Summary box**

**What is already known?**

- Injuries to law enforcement officers and firefighters have been reported throughout the literature. Still, no systematic review has been performed reporting recruit injury epidemiology, even though this population complete strenuous physical training.

**What are the new findings?**

- No studies have reported the injury profile of firefighter recruits.
- Medical attention injuries in law enforcement recruits ranged from 13.7% to 24.5%.
- Law enforcement officers’ medical attention injury incidence rate ranged from 1.67 to 4.24/1000 training days.
- Most law enforcement officers’ medical attention injuries are distributed between the upper limb (12.5%–38.2%), trunk/spine/abdomen (19.1%–50%) and lower limb (25%–41.1%).

## Background

Tactical operators (such as law enforcement officers or firefighters) undergo intense and strenuous physical training programmes as a part of their qualification process to prepare for the demands of their role, with the duration of training differing between professions and countries.^1–3^ These intense training programmes are important as they mirror the demands of the occupation and ensure law enforcement officers and firefighters are job-ready. However, these job-specific physical training programmes have been shown to result in injuries.^1–3^ Therefore, it is reasonable to expect musculoskeletal injuries to law enforcement officers and firefighters are common during their training processes.

No reviews to date have specifically explored the injury profiles of law enforcement and firefighter recruits during their academy training and physical preparation programmes.^4 5^ In firefighters, a 2019 systematic review of injury epidemiology detailed operational workplace injuries in fully qualified personnel.^4^ This review reported the proportion of injuries, ranging from 9% to 74%^4^ of participants. No studies were identified that reported injuries during prequalification recruit physical training. However, this review did exclude studies that provided interventions,^4^ potentially limiting the number of available studies and in the absence of large epidemiological studies, worth including with literature reviews.

A second systematic review reported injury occurrence in law enforcement officers, ranging from 28% to 81% of the population.^5^ However, few studies defined what was classified as an injury (eg, medical attention injury or time-loss definitions), and occupational injuries (eg, mental health concerns following a distressing work incident) were also included that do not apply to recruits in pre-deployment physical preparation programmes.^5^ As opposed to the review of firefighters, studies reporting injuries to law enforcement recruits were identified.^5^ Some of these studies involving recruits reported the definition of an injury (eg, medical attention or time-loss definitions). They demonstrated the proportion of medical attention injuries within police recruits during basic training between 15% and 26%.^3 6^ Given the sparsity of studies identified in these reviews, the inclusion of randomised controlled trials (RCTs) of injury prevention that include a standard practice (eg, natural history arm) should be considered to overcome the lack of epidemiological studies, and overcome the small samples that may lead to the imprecision of results.^7^

The challenge for clinicians and researchers who develop physical preparation and injury prevention programmes is that no reviews report how much training and preparation time is lost when firefighter and law enforcement recruits are injured during basic training, what injuries are most common, and what mechanisms of injury cause recruit injury. The nature of musculoskeletal injuries within law enforcement and firefighter recruit training are potentially more comparable to sports injuries than typical occupational injuries (eg, neck and back pain from workplace sitting)^8^ as the injuries are usually related to the fitness component of the recruits training.^9 10^ However, studies have not differentiated important components of the injury landscape potentially relevant in prevention models.^11^ Several different components related to injures can be reported: severity of injury (any injury, medical attention injury, time-loss injury or career-ending injury), relationship to activity (directly, indirectly, or not related), mode of onset (sudden or gradual), mechanism of injury (direct contact, indirect contact or non-contact), subsequent injury, body area, tissue type or pathology type.^12^ Injury data can also be presented in different ways: Injury frequency (number of injuries reported within the sample), injury proportion (percentage of different injuries within the injured participants), injury prevalence (the portion of the sample which has an injury during a specific time frame), injury incidence (the number of new injuries experienced over a specified time frame), injury incidence rate (the number of new injuries experienced when accounting for exposure), injury severity (the time-loss due to injury) or injury burden (the injury incidence combined with the injury severity).^12^ These components help inform researchers and clinicians where the ‘injury problem’ lies within their physical training programme and can help inform the development of programmes less likely to result in injury.^12^

The International Olympic Committee consensus statement on the methods for recording and reporting epidemiological data by Bahr *et al*^12^ highlights the importance of defining and classifying the health problems associated with physical activity. The reviews identified above,^4 5^ have not extracted and reported the data suggested by Bahr *et al*,^12^ which may be more meaningful for real-world translation. As an example, previous reviews have not differentiated the different injury types (eg, medical attention vs time loss) that are important for translating prevention strategies into clinical practice and policy. This enables clinicians and researchers to examine risk factors for injury and then implement prevention strategies to reduce the burden of injury. This systematic review aimed to determine the injury epidemiology of law enforcement and firefighter recruits.

## Methods

### Guidelines

The protocol for this systematic review was designed following the Preferred Reporting Items for Systematic Reviews and Meta-Analyses (PRISMA)-Protocols,^13^ with the final systematic review informed by the recent updates to the PRISMA.^14^

### Data management

Records and data related to study selection were stored online using Covidence (Covidence systematic review software, Veritas Health Innovation, Melbourne, Australia. Available at www.covidence.org). Extracted data was managed and stored using Microsoft teams and password-protected laptop computers. To facilitate systematic review transparency,^15 16^ the final data spreadsheet is also freely available (Murphy, Myles (2022): Musculoskeletal injury epidemiology in law enforcement and firefighter recruits during physical training: a systematic review. figshare. Dataset. https://doi.org/10.6084/m9.figshare.19076567.v1).

### Criteria for considering studies for this review

#### Types of studies

Prospective and retrospective studies which reported data on musculoskeletal injury were included. We included both cross-sectional and longitudinal studies (including RCTs of injury prevention interventions). For example, RCTs of an intervention within a specific injury population (eg, the effect of orthotics in police or firefighter recruits with stress fractures) were excluded, but RCTs that examined injury prevention (eg, the effect of orthotics in preventing stress fractures within police or firefighter recruits) were included provided they had a control arm without an intervention. Only published studies were included within this review (ie, grey literature excluded). Non-English language studies were also excluded. Prior work suggested that inclusion or exclusion of non-English articles do not influence the effect estimates yet may narrow CIs.^17^

#### Types of participants

We included law enforcement and firefighter recruits, regardless of sex, geographical location, age and physical activity levels.

#### Types of injuries

All musculoskeletal injuries sustained by participants were included. Injuries were defined as all medical attention and time-loss following the International Olympic Committee reporting standards.^12^ A further type of injury, an injury requiring withdrawal from the recruit training programme, was also included.

### Search methods for identification of studies

Search strategies were implemented from inception until the 5 May 2021 by a single author (MCM), who exported the records into Covidence.

#### Electronic searches

Searches were performed using free text and MESH terms (online supplemental appendix A) to identify published articles on the following electronic databases: PubMed, CINAHL, CENTRAL, SPORTDiscus and Web of Science. Only peer-reviewed, English language, human trials were included. However, these limitations were adapted to individual databases as necessary (online supplemental appendix B). Search results were piloted and validated by ensuring searches included key research papers (Orr *et al*^3^ Orr *et al* 18 and Orr *et al*^19^).

10.1136/bmjsem-2021-001289.supp1 Supplementary data

#### Searching other resources

Reference lists of relevant reviews and included studies were screened, and backwards citation tracking was performed via Web of Science to identify potentially relevant studies. Content experts evaluated the list of included studies to help identify any other relevant studies. The ePublication lists of key journals in the field (ie, journals where other included studies had been published) were screened to detect studies that had yet to be indexed in the databases.

### Selection of studies

Two review authors (H-AG/MCM or H-AG/MN) independently assessed the titles and abstracts of potential studies identified by the search strategy for their eligibility. When the study’s eligibility was unclear from the title and abstract, the full paper was assessed. Studies that did not match the inclusion criteria for this review were excluded, and the reasons for excluding full-text articles were recorded within the PRISMA flow chart.^20^ Disagreements between authors regarding study inclusion were resolved by discussion. Studies were not anonymised before assessment.

### Data management

#### Data extraction

Two review authors (H-AG/MN) independently extracted data from included studies and input the data into Microsoft Excel. For any discrepancies or disagreements, the review authors resolved these via consensus. Where consensus could not be achieved, a third author (MCM) made a majority decision after assessing the study. The following information was extracted: primary author, year of publication, country of origin, funding source, study design (retrospective or prospective data collection), study population (law enforcement or firefighter recruits), sample size (n), duration of recruit training (weeks), method of exposure to physical training (hours), mean (SD) baseline demographics (age, gender, height, weight and body mass index), all descriptive injury data inclusive of measures of variability: severity of injury, relationship to activity, mode of onset, mechanism of injury, new or subsequent injury, body area, tissue type or pathology type, and all injury data analysis inclusive of measures of variability: injury frequency, injury proportion, injury prevalence, injury incidence, injury incidence rate, injury severity and injury burden.

#### Dealing with missing data

Where a method of exposure was not provided (eg, the number of training hours was not reported), it was assumed that 1 week of recruit training represented five training exposure days. Three studies did not specify whether the injuries reported were based on the total number of injuries or the number of injured participants.^3 19 21^ To include within analysis, we assumed they reported the number of injured participants.

### Assessment of quality in included studies

Two review authors (H-AG and MN) independently assessed the quality of included studies. Where there were disagreements between review authors, they were resolved by discussion. However, where consensus could not be achieved, a majority decision was made by a third review author (MCM). The Joanna Briggs Institute, Quality Assessment Checklist for Prevalence Studies, was used to assess the study quality in the included studies.

### Assessment of diversity and heterogeneity

Given the variety in recruit training protocols (eg, differing durations or differing programmes) between studies, we had anticipated significant clinical diversity among the included populations. Total variation across all studies included within meta-analysis was planned to be explored using the I^²^ statistic, but due to substantial clinical diversity precluding meta-analysis, this was not performed.

### Assessment of reporting biases

The possible influence of publication and small study biases on review findings was considered. The influence of small study biases was addressed by the risk of bias criterion ‘study size’. Studies with fewer than 50 injuries represent a high risk of small sample bias. Studies with between 50 and 200 injuries were classified as the moderate risk of small sample bias, and studies with greater than 200 injuries were classified as low risk of small sample bias.^7^

### Data synthesis

Law enforcement and firefighter data were presented separately. Data analysis was conducted using SPSS V.27 (SPSS). All demographic data were described using mean and SD. We reported on all components of injury where data were available:

- Injury presented as a count and proportion.
- Injury prevalence was presented as a percentage over a specified time frame.
- Injury incidence was presented as the number of new injuries over a specified time frame.
- The injury incidence rate was presented as the number of injuries per measure of exposure.
- Injury severity was presented as the mean (SD) time loss.
- The injury burden was presented as the mean injury incidence multiplied by the mean injury severity (95% CIs).

All injury incidence rates were calculated as per 1000 training days (Poisson 95% CI) to allow comparisons between studies. Due to substantial clinical diversity, the limited number of studies and no studies reporting injury metrics such as severity, several planned procedures were unable to be performed, including data pooling to determine overall injury incidence, overall injury incidence rate, overall injury severity and overall injury burden with 95% CIs and meta-regression of the influence of demographic variables on the pooled effect estimates.

#### Sensitivity analysis

A sensitivity analysis had been planned but was not performed due to the limited number of studies.

#### Subgroup analysis

A subgroup analysis had been planned but was not performed due to the limited number of studies.

### Assessment of the certainty of the body of evidence

The assessment for overall certainty of the body of evidence differs in systematic epidemiological reviews compared with traditional systematic reviews of diagnostic accuracy or interventions. It can be adjusted for different models (eg, exposure).^22^ Therefore, assessment of the certainty of the body of evidence was assessed using the Grades of Recommendations, Assessment, Development and Evaluation (GRADE) approach,^23^ adapted for use in epidemiological studies.^22^ The GRADE approach involved making an overall judgement on the quality of the body of evidence-based on the overall quality with studies being upgraded or downgraded based on different factors such as the risk of bias and sample size.^23^

## Results

### Selection of studies

**Figure 1:**
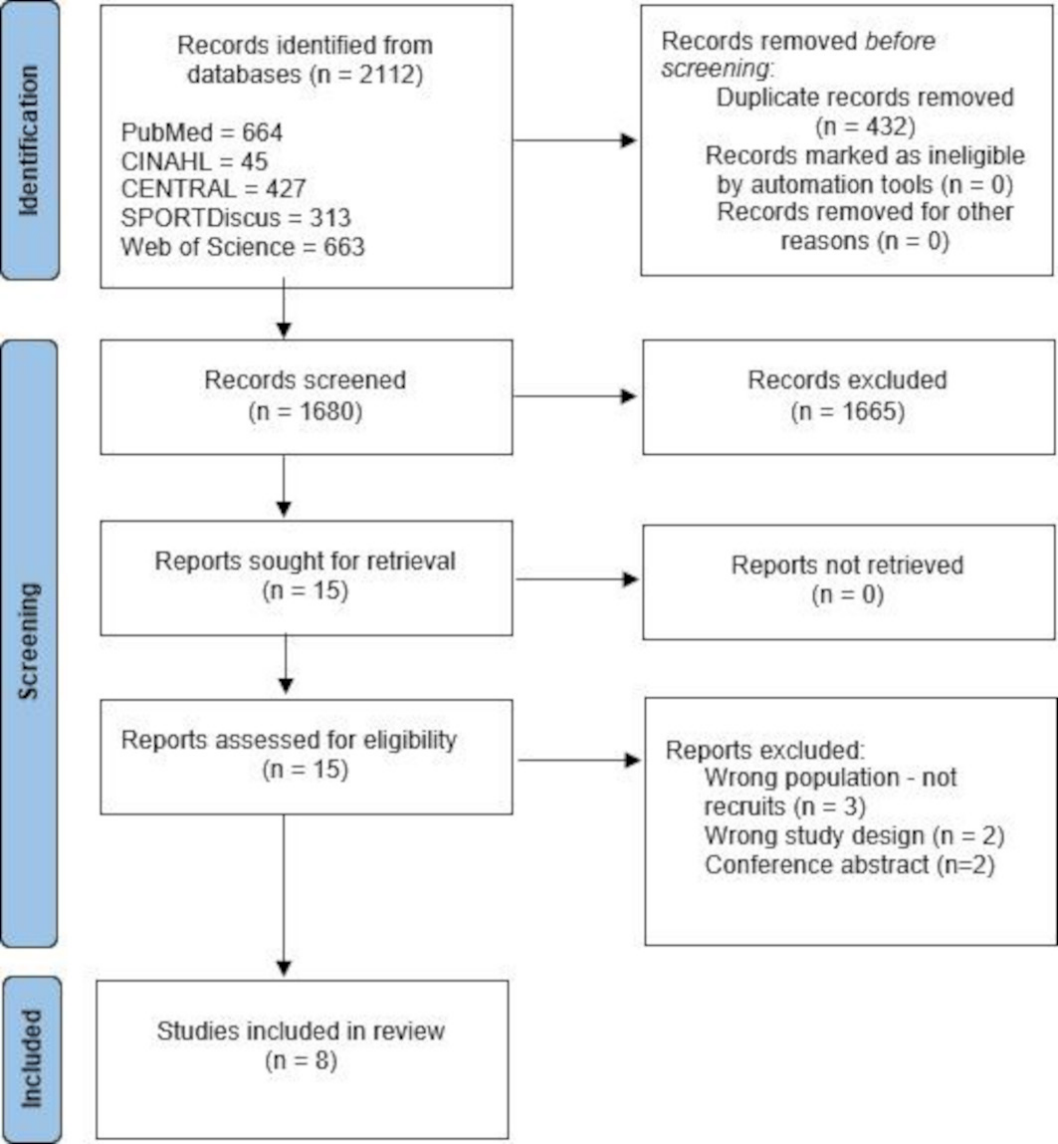
PRISMA flow chart. PRISMA, Preferred Reporting Items for Systematic Reviews and Meta-Analyses

Collectively, 2112 records were identified, eight records met the selection criteria following the full-text screening of 15 articles (figure 1). Reasons for full-text exclusion are reported in online supplemental appendix C.

### Study information

All included studies investigated injury in law enforcement recruits.^1–3 18 19 21 24 25^ No studies investigating injury in firefighter recruits were identified. Full study data are provided in table 1. Seven of the included studies (87.5%) represented a cohort study,^1–3 19 21 24 25^ whereas one study (12.5%) was a randomised trial.^18^ Three studies (37.5%) were conducted in Australia,^3 18 19^ two studies (25%) in the USA,^2 21^ one study (12.5%) in China,^25^ one study (12.5%) in Israel^1^ and one study (12.5%) in New Zealand.^24^ Six studies (75%) investigated injuries within Police officers,^3 18 19 21 24 25^ one study (12.5%) in border police^1^ and one study (12.5%) in Federal Bureau of Investigation recruits.^2^ The duration of training varied from 10 to 21 weeks.^1–3 18 19 21 24 25^ No studies reported external funding.

**Table 1:** Study information for law enforcement recruits

Study	Country	Study design	Funding source	Sampling time frame	Injury reporting (prospective/ retrospective)	Injury definition (all, medical attention, time-loss or required withdrawal from recruit training programme)	Injury nature provided (eg, fracture)	Injury region provided (eg, knee)
Constantini *et al* 2010^1^	Israel	Prospective intervention with historical control	Private	1996–2005 (control)	Prospective	Medical attention	Yes	Yes
Knapik *et al* 2011^2^	USA	Prospective cohort	Internally—Federal Bureau of Investigation and US Army Public Health Command	2009–2010	Prospective	Medical attention	Yes	No
Lockie *et al* 2019^21^	USA	Retrospective cohort	No funding	Not reported	Prospective	Withdrawal from programme	No	No
Orr *et al* 2016^3^	Australia	Retrospective cohort	Not reported	2013–2014	Prospective	Medical attention	No	No
Orr *et al* 2016^18^	Australia	Randomised controlled trial	Not reported	Not reported	Prospective	Medical attention	No	Yes
Orr *et al* 2017^19^	Australia	Retrospective cohort	No funding	2013	Prospective	Medical attention	No	No
Tomes *et al* 2020^24^	New Zealand	Retrospective cohort	Not reported	Not reported	Prospective	Medical attention	No	Yes
Wang *et al* 2003^25^	China	Retrospective cohort	Not reported	1999–2000	Prospective	Medical Attention	No	No

Seven studies (87.5%) reported medical attention injuries only^1–3 18 19 24 25^ and one study (12.5%) reported injuries resulting in discharge from the training programme.^21^ Four studies (50%) supplied some data on the region of injury. However, no studies supplied sufficient information to classify injuries according to their nature. Data collection for all studies was prospective, though medical records were obtained via the law enforcement agency database retrospectively. As the injury data for included studies were originally recorded within a database and provided to included studies on request, authors were not contacted for additional information as lack of detail was unlikely to be related to study reporting, and instead a result of database limitations.

### Participant demographics

Complete demographic details are presented in table 2. Four studies (50%) reported the sex split with a range of 0%–100% female recruits being included.^1 2 21 25^ Three studies (37.5%) reported other demographic information for participants.^1 21 25^

**Table 2:** Demographic information for law enforcement recruits

Study	Recruit type	Duration of recruit training (weeks)	Total sample size (n)	Total injured participants (n)	Total injuries (n)	Total sample mean (SD) age (years)	Total sample mean (SD) height (cm)	Total sample mean (SD) weight (kg)	Total sample mean (SD) BMI (m/kg^2^)	Total sample gender (% female)
Constantini *et al* 2010^1^	Border Police	16	1423	82	215	Not reported	Not reported	Not reported	Not reported	100
Knapik *et al* 2011^2^	Federal Bureau of Investigation	21	531	Not reported	256	Not reported	Not reported	Not reported	Not reported	20
Lockie *et al* 2019^21^	Police	Not reported	401	18	Not reported	27.3 (5.92)	174 (12)	80.27 (14.38)	Not reported	17
Orr *et al* 2016^3^	Police	12	1021	158	Not reported	Not reported	Not reported	Not reported	Not reported	Not reported
Orr *et al* 2016^18^	Police	10	287	Not reported	24	Not reported	Not reported	Not reported	Not reported	Not reported
Orr *et al* 2017^19^	Police	12	169	43	Not reported	Not reported	Not reported	Not reported	Not reported	Not reported
Tomes *et al* 2020^24^	Police	16	243	Not reported	68	Not reported	Not reported	Not reported	Not reported	Not reported
Wang *et al* 2003^25^	Police	Not reported	805	111	130	18.7 (1.02)	170.36 (5.31)	62.5 (8.8)	21.52 (2.68)	0

BMI, body mass index.

### Assessment of heterogeneity

Demographic information within studies was poorly reported, precluding judgement of whether studies were sufficiently homogenous for meta-analysis. Studies had a varied proportion of females within the sample. Law enforcement recruit training was performed in various countries for different occupations and was performed across different years/decades, suggesting that training programmes may not be similar. Meta-analysis was not conducted due to concerns regarding clinical diversity within the included samples, and statistical heterogeneity was not calculated.

### Injury profiles

#### Injury frequency and proportion

A total of 412 injured participants were reported within 3606 participants across five studies (62.5%).^1 3 19 21 25^ A total of 693 injuries were reported within 3076 participants across five studies (62.5%).^1 2 18 24 25^ Two studies (25%) reported the total number of injuries and the number of injured participants.^1 25^ Three studies (37.5%) reported the number of injured participants only.^3 19 21^ Three studies (37.5%) reported the total number of injuries only.^2 18 24^ The proportion of different injury regions was reported in two studies (online supplemental appendix D). One study of medical attention injuries in Australian police recruits reported 3/24 (12.5%) injuries occurred to the abdomen, lower back, lumbar spine and pelvis, 12/24 (50%) injuries occurred to the knee and lower leg and 6/24 (25%) injuries occurred to the ankle and foot with 3/24 injuries (12.5%) undesignated.^18^ Another study of medical attention injuries in New Zealand police recruits reported 13/68 (19.1%) injuries occurred in the trunk and spine, 26/68 (38.2%) injuries occurred in the upper limb and 28/68 (41.1%) injuries occurred in the lower limb with one injury (1.6%) not being allocated to a body region.^24^

#### Injury prevalence

The prevalence of medical attention injuries (ie, the number of injuries overall, irrespective of whether multiple injures were within a single participant) or injured participants (ie, the number of participants injured irrespective of the number of overall injuries) for the duration of their recruit training programme was provided for all studies. The prevalence of police recruits with medical attention injuries ranged from 13.7% to 24.5%.^3 19 21 25^ The prevalence of medical attention injuries within police recruits ranged from 8.4% to 27.9%.^24 25^ The prevalence of Federal Bureau of Investigation recruits with medical attention injuries was not reported.^2^ The prevalence of medical attention injuries within FBI recruits was 48.2%.^2^ The prevalence of Israeli border police recruits with stress fractures requiring medical attention was 6.8%.^1^ The prevalence of stress fractures requiring medical attention within border police recruits was 17.8%.^1^

#### Injury incidence and injury incidence rate

**Figure 2:**
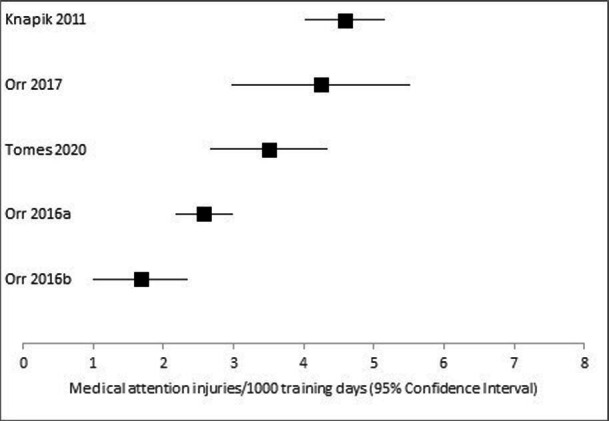
Overall medical attention injury incidence rates.

Two studies were not included within calculations of the injury incidence rates as they did not provide the duration of the recruit training programme.^21 25^ The injury incidence rates for overall medical attention injuries, injury incidence rates for medical attention injuries per body region were calculated, and injury incidence rates for stress fractures requiring medical attention were calculated. The overall medical attention injury rates are presented in figure 2. The overall medical attention injury incidence rate for police recruits ranged from 1.67 injuries per 1000 training days (Poisson 95% CI 1.00 to 2.34 injuries per 1000 training days) to 4.24 injuries per 1000 training days (Poisson 95% CI 2.97 to 5.51 injuries per 1000 training days). FBI recruits’ overall medical attention injury incidence rate was 4.59 injuries per 1000 training days (Poisson 95% CI 4.03 to 5.15 injuries per 1000 training days).

Injury incidence rates within Australian police recruits for injury regions were calculated as 0.21 abdomen, lower back, lumbar spine and pelvis injuries per 1000 training days (Poisson 95% CI 0.01 to 0.45 injuries per 1000 training days), 0.84 knee and lower leg injuries per 1000 training days (Poisson 95% CI 0.36 to 1.31 injuries per 1000 training days) and 0.42 ankle and foot injuries per 1000 training days (Poisson 95% CI 0.08 to 0.75 injuries per 1000 training days). The injury incidence rates within New Zealand police recruits for injury regions were calculated as 0.67 trunk and spine injuries per 1000 training days (Poisson 95% CI 0.31 to 1.03 injuries per 1000 training days), 1.34 upper limb injuries per 1000 training days (Poisson 95% CI 0.82 to 1.85 injuries per 1000 training days) and 1.44 lower limb injuries per 1000 training days (Poisson 95% CI 0.91 to 1.97 injuries per 1000 training days). The injury incidence rates for stress fractures in Israeli border police recruits were calculated as 2.22 stress fractures per 1000 training days (Poisson 95% CI 1.92 to 2.52 injuries per 1000 training days).

#### Injury severity and burden

One study in US police recruits reported that 18/401 (4.5%) recruits were discharged from the training programme due to injuries. No other studies reported on the severity or burden of injury.^21^

### Assessment of quality in included studies

The overall quality for each study was assessed as low (table 3).^1–3 18 19 21 24 25^ Two studies were low quality for the sample frame as they were greater than 10 years old and unlikely to represent current populations.^1 25^ Two studies were assessed as unclear quality due to sample size. They did not report the number of participants who had injuries^2 24^ and three studies were assessed as low quality as they had fewer than 50 injured participants.^3 19 21^ All studies were judged as low quality for describing the subject and setting as no study presented participant age, height, weight, and the training programme.^1–3 18 19 21 24 25^ Statistical analysis was considered not applicable as we purely extracted injury numbers. The response rate was also considered not applicable as all studies used a database to collect prospective injury data and later sourced these medical records.

**Table 3:** Quality of included studies

Study	1. Was the sample frame appropriate to address the target population?	2. Were study participants sampled in an appropriate way?	3. Was the sample size adequate?	4. Were the study subjects and the setting described in detail?	5. Was the data analysis conducted with sufficient coverage of the identified sample?	6. Were valid methods used for the identification of the condition?	7. Was the condition measured in a standard, reliable way for all participants?	8. Was there appropriate statistical analysis?	9. Was the response rate adequate, and if not, was the low response rate managed appropriately?	Overall judgement
Constantini *et al* 2010^1^	Low	High	High	Low	High	High	High	Not applicable	Not applicable	Low
Knapik *et al* 2011^2^	High	High	Unclear	Low	High	High	High	Not applicable	Not applicable	Low
Lockie *et al* 2019^21^	High	High	Low	Low	High	Unclear	High	Not applicable	Not applicable	Low
Orr *et al* 2016^3^	High	High	Low	Low	Unclear	High	High	Not applicable	Not applicable	Low
Orr *et al* 2016^18^	High	High	High	Low	Unclear	High	High	Not applicable	Not applicable	Low
Orr *et al* 2017^19^	High	High	Low	Low	Unclear	High	High	Not applicable	Not applicable	Low
Tomes *et al* 2020^24^	High	High	Unclear	Low	High	High	High	Not applicable	Not applicable	Low
Wang *et al* 2003^25^	Low	High	High	Low	High	High	High	Not applicable	Not applicable	Low

### Assessment of the certainty of the body of evidence

Injury incidence rates were based on data extracted from individual studies (number of injuries and the duration of the recruit training programme). However, the certainty of the injury incidence rates presented within this systematic review was judged to be very low, suggesting that the true injury incidence rate may be substantially different. The certainty of the evidence was downgraded as all studies were of low quality,^1–3 18 19 21 24 25^ indirectness (some studies did not appear representative of the target population, and all studies failed to present sufficient demographic and exposure data^1–3 18 19 21 24 25^ and inconsistency (the 95% CIs of the injury incidence rate, within figure 2, did not overlap in all studies).

## Discussion

This systematic review identified eight studies that presented injury data within law enforcement officers. Unfortunately, we were unable to identify any studies which reported injury epidemiology within firefighter recruits, of the eight studies assessing injury epidemiology in law enforcement officers, seven defined injuries using a medical attention definition and one defined injury as career-ending. Therefore, this review provides insight into the epidemiology of medical attention and retirement injuries in law enforcement officers. However, we could not identify any studies reporting all injuries or time-loss injuries within law enforcement recruits.

All studies reported the number of overall injuries or the number of injured recruits; however, only two studies reported both. The lack of reporting the number of injuries and number of injured participants by 75% of studies limited the sample size to calculate injury frequency and injury proportion, decreasing confidence in the estimate. Only two studies, both in police officers, provided sufficient detail to report the injury incidence of the body region.^18 24^ One aim of injury monitoring is to identify the injuries which end up being the most significant^26^ as far as time-loss for the recruit, resource allocation for the organisation and being of the largest financial expense. However, only one study reported on the severity and burden of injury.^21^ Even more limiting is that this study did not provide the injury region or injury nature, limiting the design specificity of injury risk reduction programmes. All studies included within this review obtained injury reports retrospectively from the partner organisation database, which had collected data prospectively. While this design removes the influence of recall bias, typically seen within retrospective studies,^27^ it does mean that injury data provided is limited to that routinely collected by the organisation. This means the capacity to report various components of injury is not possible unless already collected by the organisation.

Injury prevention interventions for law enforcement officers would appear to require a programme targeting upper limb, spinal and lower limb injury given the distribution of injury regions. However, no studies have reported injury severity and injury burden. Further research into the injuries that are most costly to law enforcement officers, and their respective organisations is recommended prior to the development of prevention interventions. Thus, ensuring prevention interventions target those injuries associated with the largest injury burden.

The sparsity of data on injuries to firefighter recruits and data related to all injury and time-loss injury in law enforcement recruits was surprising given the number of recruits trained internationally and the financial burden associated with injured recruits. According to a 2014–15 Australian police report, the average cost to train a police recruit is $A84 000.^28^ Therefore, based on the data from Lockie *et al*,^21^ we extrapolated that the cost of 18/401 recruits leaving the programme due to injury would cost upwards of $A350 000/100 recruits commencing a training programme. Based on an annual report from one of eight Australian states or territories, 150 additional police recruits were to be recruited over the 2020–2021 calendar year^29^ that, based on the estimates above, could result in a financial loss of over half a million $A due to recruits leaving the programme due to injury.

In a sports injury setting, athletes are screened for injury risk factors. An injury risk reduction programme is implemented to eliminate these risk factors, subsequently reducing the burden of injuries.^30 31^ However, according to the Translating Research into Injury Prevention Practice framework, an important step in reducing injuries is by accurate injury epidemiology.^26^ Our review has identified that no studies have reported the injury epidemiology of firefighter recruits and that the certainty of the evidence detailing the injury epidemiology of law enforcement recruits is very low. Therefore, further investigation of the injury epidemiology within firefighter and law enforcement recruit populations is needed before developing meaningful injury risk reduction interventions.

### Limitations

All studies included within this review reported injuries documented as medical records, which means that data are limited to what is reported to the employer (eg, the Police or FBI) during recruit training. We would suggest that for future injury epidemiology studies investigating law enforcement and firefighter recruits, data collection is planned prospectively and includes more detailed injury and exposure data (such as that suggested by Bahr *et al* for sports injury populations)^12^ but is also conscious of the burden of reporting on key stakeholders.^32^ This would involve reporting the mechanism of injury (eg, running or resistance training), further breaking down injuries into regions (such as the injury regions provided by the International Classification of Disease), reporting the type of injury (eg, tendon injury vs bone injury).^12^ We would also suggest future studies provide more in-depth analysis inclusive of other injury metrics such as injury severity and burden to inform which injuries result in the most time lost from recruit training.

As detailed within the methods, no studies provided a measure of training exposure (eg, the number of training hours was not reported), so it was assumed that 1 week of recruit training represented five training exposure days for calculating the injury incidence rate. Without an accurate measure of training exposure, the assumption that 1 week of training represented five training days may not be accurate. Additionally, when a study did not specify whether the injuries reported were based on the total number of injuries or the number of injured participants, to include within incident rate analysis, it was assumed they reported the number of injured participants. Future studies should consider including training exposure and more clarity about new vs subsequent/recurrent injuries to enable a more accurate calculation of incidence rates.

### Conclusion

This review could not identify any studies reporting the injury epidemiology of firefighter recruits. This review was able to identify eight published studies that reported the injury epidemiology of law enforcement recruits. However, the studies were all of low quality, and the credibility of the evidence was assessed as very low. Seven studies reported medical attention injuries, and one study reported the number of medical withdrawals from a recruit training programme. The prevalence of police recruits with medical attention injuries ranged from 13.7% to 24.5%. The overall medical attention injury incidence rate for police recruits ranged from 1.67 injuries per 1000 training days (Poisson 95% CI 1.00 to 2.34 injuries per 1000 training days) to 4.24 injuries per 1000 training days (Poisson 95% CI 2.97 to 5.51 injuries per 1000 training days). No studies reported on the severity or burden of injuries.
