# Supplementary material for: Musculoskeletal injury epidemiology in law enforcement and firefighter recruits during physical training: a systematic review
Source: BMJ Open Sport Exerc Med. 2022 Mar 1;8(1):e001289. doi: 10.1136/bmjsem-2021-001289 (PMC8889355; doi:10.1136/bmjsem-2021-001289)

Appendix A. Systematic Review Search Strategy

**Number**	**Combiners**	**Terms**
1	Problem of Interest	Injur*
2	Participants	Recruit* OR trainee*
3	Participants	fire fight* OR first respon* OR emergency respon* OR police OR law enforce*
4	Exclusion	review OR meta-analysis
5		#1 AND #2 AND #3 NOT #4
	Limitations	Peer reviewed, human, English language,

Appendix B. Search Strategy Documentation

Source:	Date of search	Search strategy used (keywords & Boolean)	Search Limits or filters (e.g. dates, language)	# results found	Comments
PUBMED	05/05/2021	(((Injur*) AND (Recruit* OR trainee*)) AND (fire fight* OR first respon* OR emergency respon* OR police OR law enforce*)) NOT (review OR meta-analysis)	Human, English	664	Exported to End Note
CINAHL (Full Text)	05/05/2021	(((Injur*) AND (Recruit* OR trainee*)) AND (fire fight* OR first respon* OR emergency respon* OR police OR law enforce*)) NOT (review OR meta-analysis)	Peer reviewed, human, English	45	Exported to End Note
CENTRAL	05/05/2021	(Injury) AND (Recruit OR trainee) AND (fire fighter OR first response OR emergency response OR police OR law enforcement)	Word variations, trials	427	Exported to End Note
SportsDISCUS	05/05/2021	(((Injur*) AND (Recruit* OR trainee*)) AND (fire fight* OR first respon* OR emergency respon* OR police OR law enforce*)) NOT (review OR meta-analysis)	Peer-reviewed, English	313	Exported to End Note
Web of Science	05/05/2021	(((AB= Injur* AND AB=(Recruit* OR trainee*) AND AB=(fire fight* OR first respon* OR emergency respon* OR police OR law enforce*)) NOT ALL=(review OR meta-analysis)))	English, article	663	Exported to End Note
Key Journals					N/A
				TOTAL	2112

Appendix C. Reasons for full-text exclusion

Study	Title	Reason for exclusion
Mann et al. 2008	Stress fractures in female border police recruits during basic training	Conference abstract
Mann et al. 2008	Stress fractures reduction by equipment modification in border police female fighters recruits: part II	Conference abstract
Mitrovic et al. 2016	How an eight-month period without specialized physical education classes affects the morphological characteristics and motor abilities of students of the academy of criminalistic and police studies.	Wrong study design
Mostardi et al. 1990	Pre-employment screening and health management for safety forces - methods and techniques	Wrong patient population (not recruits)
Orr et al. 2016	A functional movement screen profile of an Australian state police force: a retrospective cohort study	Wrong study design
RuZBarskA et al. 2010	Analysis of motor performance indicators in medical rescuers	Wrong patient population (not recruits)
Thabouillot et al. 2017	Medical causes of temporary or definitive leaves from a French counterterrorist unit pre-internship	Wrong patient population (not recruits)

Appendix D. Medical injuries according to body region.

i) Orr et al. 2016b

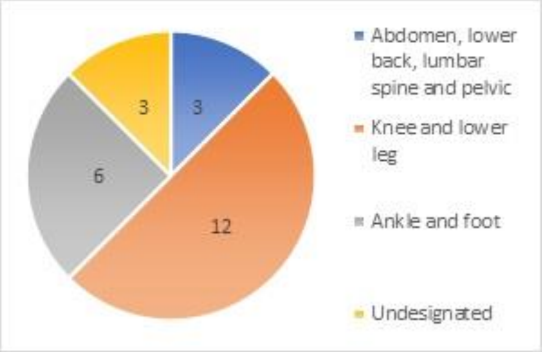

ii) Tomes et al. 2020

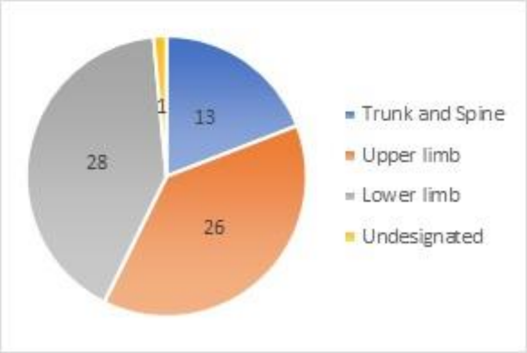

Supplement: Supplementary data [file bmjsem-2021-001289supp001.pdf]
